# An approach to the immunophenotypic features of circulating CD4^+^NKG2D^+^ T cells in invasive cervical carcinoma

**DOI:** 10.1186/s12929-015-0190-7

**Published:** 2015-10-20

**Authors:** Mariel Garcia-Chagollan, Luis Felipe Jave-Suarez, Jesse Haramati, Miriam Ruth Bueno-Topete, Adriana Aguilar-Lemarroy, Ciro Estrada-Chavez, Blanca Estela Bastidas-Ramirez, Ana Laura Pereira-Suarez, Susana del Toro-Arreola

**Affiliations:** Departamento de Fisiología, Laboratorio de Inmunología, CUCS, Universidad de Guadalajara, Sierra Mojada # 950, Guadalajara, Jalisco México; División de Inmunología, CIBO, Instituto Mexicano del Seguro Social, Guadalajara, Jalisco México; Departamento de Biología Celular y Molecular, Laboratorio de Inmunobiología, CUCBA, Universidad de Guadalajara, Guadalajara, Jalisco México; Departamento de Biología Molecular y Genómica, Instituto de Enfermedades Crónico-Degenerativas, CUCS, Universidad de Guadalajara, Guadalajara, Jalisco México; Centro de Investigación y Asistencia en Tecnología y Diseño del Estado de Jalisco, Unidad de Biotecnología Médica y Farmacéutica, Guadalajara, Jalisco México

**Keywords:** NKG2D, CD4^+^NKG2D^+^ T cells, Cervical carcinoma

## Abstract

**Background:**

NKG2D, an activating immunoreceptor, is primarily restricted to NK cells and CD8^+^ T cells. The existence of an atypical cytotoxic CD4^+^NKG2D^+^ T cell population has also been found in patients with autoimmune dysfunctions. Nonetheless, contradictory evidence has categorized this population with a regulatory rather than cytotoxic role in other situations. These confounding data have led to the proposal that two distinct CD4^+^NKG2D^+^ T cell subsets might exist. The immune response elicited in cervical cancer has been characterized by apparent contradictions concerning the role that T cells, in particular T-helper cells, might be playing in the control of the tumor growth. Interestingly, we recently reported a substantial increase in the frequency of CD4^+^NKG2D^+^ T cells in patients with cervical intraepithelial neoplasia grade-1. However, whether this particular population is also found in patients with more advanced cervical lesions or whether they express a distinctive phenotype remains still to be clarified. In this urgent study, we focused our attention on the immunophenotypic characterization of CD4^+^NKG2D^+^ T cells in patients with well-established cervical carcinoma and revealed the existence of at least two separate CD4^+^NKG2D^+^ T cell subsets defined by the co-expression or absence of CD28.

**Results:**

Patients with diagnosis of invasive cervical carcinoma were enrolled in the study. A group of healthy individuals was also included. Multicolor flow cytometry was used for exploration of TCR alpha/beta, CD28, CD158b, CD45RO, HLA-DR, CD161, and CD107a. A Luminex-based cytokine kit was used to quantify the levels of pro- and anti-inflammatory cytokines. We found an increased percentage of CD4^+^NKG2D^+^ T cells in patients with cervical cancer when compared with controls. Accordingly with an increase of CD4^+^NKG2D^+^ T cells, we found decreased CD28 expression. The activating or degranulation markers HLA-DR, CD161, and CD107a were heterogeneously expressed. The levels of IL-1beta, IL-2, TNF-alpha, and IL-10 were negatively correlated with the percentages of CD4^+^NKG2D^+^ T cells in patients with cervical carcinoma.

**Conclusions:**

Taken together, our results reveal the existence of two separate CD4^+^NKG2D^+^ T cell subsets defined by the co-expression or absence of CD28, the latter more likely to be present in patients with cervical cancer.

## Background

Why do some CD4^+^ T cells exhibit cytotoxic behaviour? This has been an important question deciphered, in part, by the fact that these cells express activating receptors mostly confined to lymphocytes, which by nature are armed with a cytotoxic license [1–3]. More recent evidence over the last years has revealed the expression of a central innate cytotoxicity receptor on a small population of CD4^+^ T cells; such is the case of the immunoreceptor NKG2D [4–7]. This receptor is highly conserved in primates and rodents, and its symmetric structure in humans consists of a homodimer assembled to adaptor molecules, such as DAP10 [8, 9]. NKG2D/DAP10 signaling is facilitated via phosphatidylinositol-3 kinase (PI3K), leading to the activation of mechanisms involved in cell survival, cytoskeletal rearrangement, and release of cytotoxic granules [10–12]. That is how NKG2D after recognizing a plethora of ligands, which are for instance, largely up-regulated by actively growing epithelial tumors, confers a significant activating (NK cells) or costimulating (cytotoxic T lymphocytes) signal to trigger the mechanisms already mentioned above [13–17].

CD4^+^ T cells have been generally pre-defined as helpers and regulators of the immune responses [18–20]. However a potential cytotoxic role within this population has come to be discovered by the particular existence of an unusual small subset comprised by CD4^+^ T cells that do express NKG2D, which could represent a particular cytotoxic population involved in viral infections and chronic diseases. At this point, Groh *et al.* reported a substantial number of peripheral and synovial CD4^+^CD28^−^ T cells, which expressed the activating receptor NKG2D in patients with rheumatoid arthritis; moreover, this population promoted the cytotoxic damage against synoviocytes with anomalous expression of NKG2D ligands [21]. The existence of a large proportion of CD4^+^NKG2D^+^ T cells has also been reported in HTLV-1-associated neurologic disease, as well as in human cytomegalovirus-seropositive individuals [4, 22]. Paradoxically, it has also been reported the existence of a normally-occurring CD4^+^NKG2D^+^ T cell population, apparently endowed with regulatory activity in healthy individuals, and interestingly the expansion of this population appeared to be inversely correlated with disease severity in patients with juvenile-onset systemic lupus suggesting a regulatory rather than cytotoxic role [23]. Furthermore, in other studies involving samples from patients with different malignancies, it was noted that a large proportion of CD4^+^NKG2D^+^ T cells with regulatory activity was largely dependent of FasL and NKG2D ligands [24], supporting the idea that an immunosuppressive property distinguishes these cells in some circumstances, such as cancer. The question, which then arises, is whether this particular population would be favouring an anti-tumor immune response, or otherwise would be facilitating the tumor growth.

In particular, cervical cancer, which still remains as one of the most common malignant tumors among women in the developing world [25, 26], is predominantly controlled by functional cellular immunity under the action of both CD4^+^ and CD8^+^ T cells [27, 28]; however, these tumors have been characterized by apparent contradictions in the immune response [29–32], which could be partially explained by the action of specific CD4^+^ T cell subsets. All of this is further complicated by the apparent existence of the two functionally distinct CD4^+^NKG2D^+^ T cell subpopulations, which could influence the fate of the anti-tumor immune response, that is, to support cytotoxicity *versus* immunoregulation or *vice versa*. Compelling evidence by our group recently demonstrated an increase of CD4^+^NKG2D^+^ T cells in patients with low-grade cervical lesions, even when overall levels of CD4^+^ T cells did not increase. Also, in that study it was found that while TGF-β1 was significantly decreased in patients compared to healthy donors, both TNF-α and IL-15 showed a tendency to increase [33]. Thus, with these findings we believe that the expansion of CD4^+^NKG2D^+^ T cells in premalignant cervical lesions might be under the control of factors that are not well characterized at the moment, and that this population might be also expanded in established cancerous lesions of uterine cervix. However, the immunophenotypic signature characterizing these cells in cervical cancer patients has not yet been well defined. Therefore, the focus of our present work was to determine whether the population of NKG2D-expressing CD4^+^ T cells is also increased in invasive cervical carcinoma and to characterize the phenotypic profile of this particular population. Our present results reveal the existence of two separate CD4^+^NKG2D^+^ T cell subsets defined by the co-expression or absence of CD28, which might indicate a functional dichotomy within this particular subset. On the other hand, the negative and significant correlation between the levels of IL-1β, IL-2, TNF-α, and IL-10 and the percentages of CD4^+^NKG2D^+^ T cells might suggest that increased levels of these cells are more likely to be present in anti-inflammatory environments in patients with cervical cancer.

## Methods

### Patients and healthy donors

We recruited 31 patients that were first subjected to conventional colposcopic evaluation and finally diagnosed as invasive squamous cervical carcinoma by histopathology results. Thirty-one age/gender matched healthy donors without histories of abnormal Pap smears were included. All patients were attended at the Instituto Jalisciense de Cancerología, OPD (Guadalajara, México) and Centro Médico Nacional de Occidente, IMSS (Guadalajara, México). Prior to be enrolled, the purpose and procedures of the study were explained to patients and informed consent was obtained from each patient or healthy control. Clinical and epidemiological features were also obtained.

### Ethical considerations

All samples were acquired according to the guidelines of the Ethics Committee of the Centro Universitario de Ciencias de la Salud, Universidad de Guadalajara (Guadalajara, Mexico), in accordance with the official guidelines (Norma Oficial Mexicana, NOM) and the World Medical Association Declaration of Helsinki. All women were informed of their rights, the goals of the study, and the importance of their participation. The procedures used for collection of samples were identical to those routinely used in the clinical setting and used for patients not being part of this study.

### Collection of specimens

Five mL of peripheral blood (PB) was collected in tubes containing EDTA buffer and processed for multicolor flow cytometry analyses within 2 h after arrival at the laboratory. The remaining peripheral blood was centrifuged at 300 g for 15 min in order to obtain the plasma, which was stored at –70 °C until processing by ELISA assays.

### Antibodies

Fluorochrome-labeled monoclonal primary antibodies for simultaneous multi-antibody staining were purchased from BioLegend Inc. (San Diego, CA, USA) and the list included: PE/Cy7 anti-human CD3 (clone UCHT1), PerCP/Cy5.5 anti-human CD4 (clone RPA-T4), PE anti-human NKG2D (clone 1D11), APC anti-human CD28 (clone CD28.2), APC anti-human TCR αβ (clone IP26), APC anti-human TCR Vα24Jα18 (iNKT cell) (clone 6B11), APC anti-human CD107a (clone H4A3), FITC anti-human CD158b (clone DX27), FITC anti-human CD45 RO (clone UCHL1), FITC anti-human CD161 FITC (clone HP-3G10), FITC anti-human HLA-DR (clone L243). Isotype control antibodies were also purchased from BioLegend Inc.

### Immunophenotyping of CD4^+^NKG2D^+^ T cells by multicolor flow cytometry

Multicolor flow cytometric analysis was performed to detect the frequency and immunophenotypic profile of the CD4^+^NKG2D^+^ T cell population. Assay tubes with 100 μL of freshly isolated whole blood from cervical cancer patients or healthy controls was stained with a mixture of the corresponding antibodies for 45 min at 4 °C. After incubation, erythrocytes were lysed using BD FACS Lysing Solution (BD Bioscience, San Jose, CA, USA). The expression of TCR αβ, TCR Vα24Jα18, CD28, CD45RO, CD158b, CD107a, CD161 and HLA-DR was detected on the gate drawn around the CD3^+^CD4^+^NKG2D^+^ lymphocyte population. Firstly, lymphocytes were distinguished from other mononuclear cells using morphological parameters. Then, gate was drawn around the lymphocyte population, which was then analyzed separately using the labeling antibody scheme. Attune® NxT Acoustic Focusing Cytometer (Life Technologies, Carlsbad, CA, USA) was used to acquire twenty thousand events in the lymphocyte region. FACS data were analyzed using FlowJo software, version 8.7 (Tree Star, Inc., Ashland, OR, USA).

### Cytokine quantification

A commercial magnetic bead-based immunoassay (Bio-Rad Laboratories Inc., Hercules, CA, USA) enable to quantify multiple cytokines in a single well was used to quantify the cytokines in plasmas from cervical cancer patients or control women. Briefly, we designed a kit, which included the detection of pro- and anti-inflammatory cytokines: IL-15, IL-1β, IL-6, IL-17, TNF-α, IFN-γ, IL-2, and IL-10. Thawed samples, standard dilution series, and blanks were pipetted into designated wells; then plate was incubated at RT for 30 min, at 300 rpm shaking to enhance detection assay. After a series of washes to remove unbound protein, a biotinylated detection antibody was added to create a sandwich complex. The final detection complex was formed with the addition of streptavidin-phycoerythrin conjugate. Data were acquired using a Bio-plex system reader and management of results was performed using Bio-Plex Data Pro software.

### Statistical analysis

All data were tested for normal distribution using Kolmogorov-Smirnov test. Due to non-normal distribution Mann–Whitney *U*-test was used to compare two groups of analysis (healthy donors *vs* cervical cancer patients). Based on our previous results we stratified our present results in three different ranges (0–2, 2–4, and >4 % of CD4^+^NKG2D^+^ T cells). Corresponding to these ranges, results of CD4^+^NKG2D^+^ T cells were tested using Pearson’s chi-square test and data were expressed as percentage of frequency. According to this stratification we performed a subsequent analysis to evaluate the expression of different markers expressed by CD4^+^NKG2D^+^ T cells. In order to measure statistical dependence between the variables, Spearman’s rank correlation coefficient was used. All the statistical analyses were performed considering *p* ≤ 0.05 to be significant using SPSS 15.0 software (SPSS, Chicago, IL, USA).

## Results

### High numbers of CD4^+^NKG2D^+^ T cells are circulating in patients with established cervical carcinoma

**Fig. 1:**
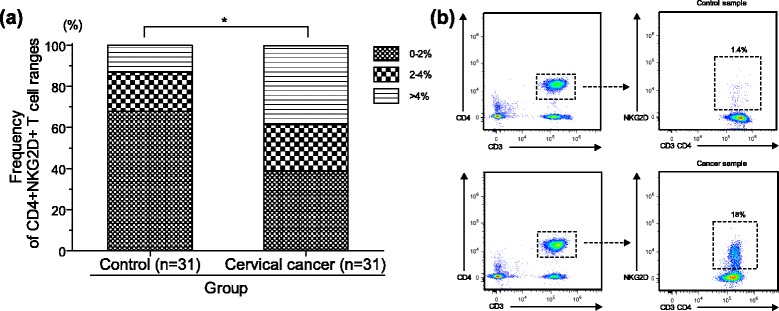
Frequency of circulating CD4^+^NKG2D^+^ T cells in patients with established cervical carcinoma. Multicolor flow cytometric analysis was performed to measure the expression of NKG2D on peripheral CD4^+^ T cells from both healthy donors and cervical cancer patients. Lymphocytes were gated according to FS and SS characteristics and sub-gated around CD3^+^ and CD4^+^ T cells; subsequently, NKG2D expression was evaluated. Bars in (**a**) represent the distribution of samples with varying levels of CD4^+^NKG2D^+^ T cells within the control and patient groups. Based on previous results, here our data were stratified in three different ranges of CD4^+^NKG2D^+^ T cells (0–2, 2–4, and >4 %). A significant increase in the frequency of CD4^+^NKG2D^+^ T cells in the group of cervical cancer patients was found, while some 68 % of the different control samples expressed very low CD4^+^NKG2D^+^ T cells (0–2 %) (*χ* ^2^(2) = 6.531; *p* = 0.038). A representative example of this disparity is shown in (**b**), where 18 % of the cancer patient’s CD3^+^CD4^+^ T cells are positive for NKG2D (lower panel), while only 1.4 % of the healthy control’s CD3^+^CD4^+^ T cells show this staining (upper panel)

We have previously reported an increase of CD4^+^NKG2D^+^ T cells in patients with low-grade cervical intraepithelial neoplasia, which might be the result of a chronic exposure to viral and/or pro-inflammatory factors [33]. In this context, different groups have addressed the effect of host pro-inflammatory responses in human papillomavirus (HPV)-related diseases [29, 34], which might promote lesion progression to more advanced stages and affect tumor fate by diverse mechanisms including the direct participation of unusual immune cells, as would be the case of the CD4^+^NKG2D^+^ T cells. Here, we wanted to focus our attention and search for this T cell population in peripheral blood from patients with cervical carcinoma. Thirty-one patients with established histopathological diagnosis of invasive squamous cervical carcinoma were enrolled in this study (ages ranged from 31–77 years, with a mean age of 51.45 years); additionally, a control group of 31 donors matched in age/gender was also included (ages ranged from 38–70 years with a mean age of 50.19 years). First, lymphocytes were gated according to their forward scatter (FS) and side scatter (SS) characteristics. Then, further gates were placed around those CD3^+^ and CD4^+^ T cells, and subsequently the NKG2D expression was evaluated. Based on our own results and also reported by others [5, 33], we have frequently seen that the CD4^+^NKG2D^+^ T cell population is not higher than 2 % in healthy controls, and that expansions of this population are often seen in patients with premalignant cervical lesions. Taking into account these data, we stratified our current results in three different ranges of CD4^+^NKG2D^+^ T cells (0–2, 2–4, and >4 %). Patient samples were significantly more likely to have higher levels of CD3^+^CD4^+^NKG2D^+^ T cells than control samples. While present to some extent in all samples tested, the distribution of this particular T cell population is significantly increased in cervical cancer patients (*χ*^2^(2) = 6.531; *p* = 0.038). In Fig. 1a we see that some 68 % of the different control samples expressed very low CD4^+^NKG2D^+^ T cells (0–2 %). Only 13 % of the control samples expressed CD4^+^NKG2D^+^ T cells above the level of 4 %, while 39 % of cervical cancer patient samples had levels of 4 % or greater of these cells. A representative example of this disparity is shown in Fig. 1b, where 18 % of the cancer patient’s CD3^+^CD4^+^ T cells are positive for NKG2D (lower panel), while only 1.4 % of the healthy control’s CD3^+^CD4^+^ T cells show this staining (upper panel).

### Circulating CD4^+^NKG2D^+^ T cells in patients with established cervical cancer express the conventional TCR αβ^+^ heterodimer

**Fig. 2:**
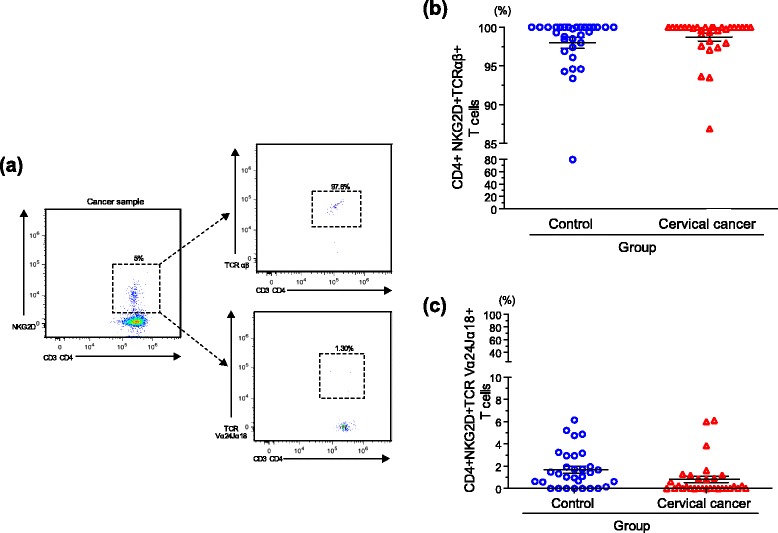
CD4^+^NKG2D^+^ T cells express the conventional TCR αβ in both healthy donors and cervical cancer patients. Multicolor flow cytometry analysis was performed in order to discard that the triple positive CD3^+^CD4^+^NKG2D^+^ T cells were either γδ T cells or iNKT cells. In (**a**) we show a representative example of high percentage of CD4^+^ T cells expressing NKG2D in a cervical cancer sample. Following multicolor staining to detect CD3 expression and CD4 co-expression, NKG2D was evaluated. Then, expression of TCR αβ and Vα24Jα18 (in order to know whether they represented a population of iNKT cells) was measured on the triple positive CD3^+^CD4^+^NKG2D^+^ T cell population. In (**b**) we show the distribution of TCR αβ on CD4^+^NKG2D^+^ T cells in controls and cervical cancer patients. More than 95 % of the CD3^+^CD4^+^NKG2D^+^ T cells were TCR αβ^+^ in both controls and cervical cancer patients. Distribution of TCR Vα24Jα18 on selected CD3^+^CD4^+^NKG2D^+^ T cells in controls and cervical cancer patients is shown in (**c**), which indicates that CD3^+^CD4^+^NKG2D^+^ T cells were mostly negative to Vα24Jα18 in both groups. Data are expressed as mean ± SEM

Given that invariant NKT cells can be defined by phenotype, *e.g.* CD4^+^*vs* CD4^−^ [35], it is possible that CD4^+^ NKT cells may be expanded within the total CD4^+^NKG2D^+^ T cell population. Similar to NKT cells, other unconventional innate-like cells, such as γδ T lymphocytes may also contribute to the total pool of T cells expressing the immunoreceptor NKG2D. Thus, in order to discard the possibility that the triple positive CD3^+^CD4^+^NKG2D^+^ cells observed were either γδ T cells or invariant NKT cells, we stained with antibodies specific for αβ TCR and Vα24Jα18 TCR α chain. Figure 2a shows a representative sample in which the double positive CD3 and CD4 lymphocyte population was sub-gated based on NKG2D expression. Then, the expression of αβ TCR and Vα24Jα18 TCR α chain was explored. As expected, we found that greater than 95 % of the CD3^+^CD4^+^NKG2D^+^ cells were TCR αβ^+^ (Fig. 2b) and fewer than 2 % of these cells were positive for the common invariant chain Vα24Jα18 (Fig. 2c). The blood from the control samples tended to have almost double the amount of invariant chain positive cells, but this result was not significant.

### CD28 costimulatory receptor defines the existence of different CD4^+^NKG2D^+^ T cell populations in established cervical carcinoma

**Fig. 3:**
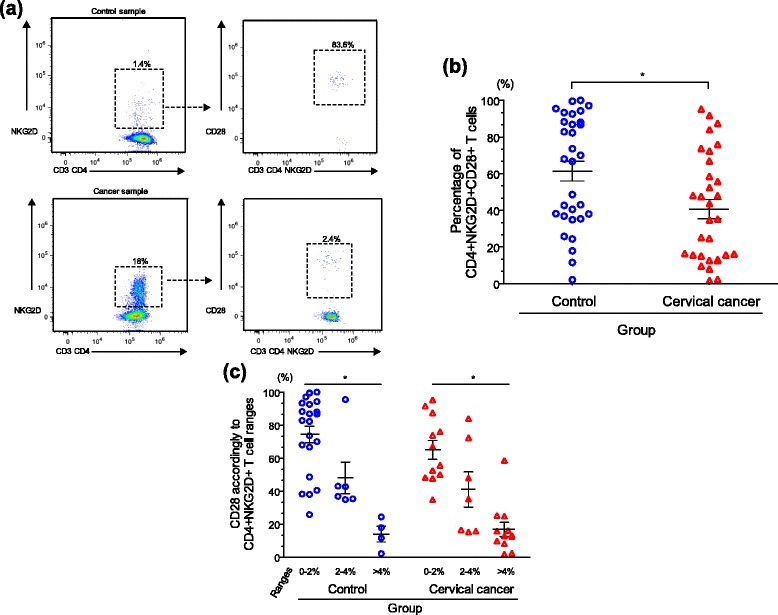
CD28 expression defines two different populations of CD4^+^NKG2D^+^ T cells in cervical cancer patients. Flow cytometry analysis of multicolor staining was performed in order to detect the expression of CD28 on selected CD3^+^CD4^+^NKG2D^+^ T cells. In (**a**) representative experiments show the expression of CD28 in a control sample with low percentage of CD4^+^NKG2D^+^ T cells (upper panel) and cancer sample with high percentage of CD4^+^NKG2D^+^ T cells (lower panel). In (**b**) we see a significant difference between patient and control samples with control CD4^+^NKG2D^+^ T cells significantly more likely to express CD28 (Mann–Whitney *U*-test; *p* = 0.008). In (**c**) patient and control samples were divided into groups based on percentage of CD4^+^NKG2D^+^ T cells and we see a division, with the loss of CD28 in both patients and controls that have high numbers of CD4^+^NKG2D^+^ T cells. Negative and statistically significant correlations between CD28 expression and percentages of CD4^+^NKG2D^+^ T cells are seen in both control (Spearman ρ = -0.522; *p* = 0.003) and patient (Spearman ρ = -0.859; *p* < 0.001) groups when segregated based on percentage of CD4^+^NKG2D^+^ T cells

In human CD8^+^ T cells, it has been reported that the immunoreceptor NKG2D serves as a costimulatory platform for TCR-mediated signaling through the recruitment of the p85 regulatory subunit of PI3K, in a manner similar to CD28 [15]. Recently, a functional dichotomy for both CD28 and NKG2D in distinct CD8^+^ T cells has been noted [36, 37]. In our current work, we find that, within the CD3^+^CD4^+^NKG2D^+^ T cell gate, it is the presence or absence of CD28 that defines at least two cell populations, both present in patient and control samples. CD28 expression was evaluated on selected lymphocytes, which were previously gated on CD3 and CD4 double-positive cells, and then sub-gated on NKG2D co-expression as shown in Fig. 3a, which depicts representative analyses of these measurements.

We see that many of the patient and control samples had a substantial percentage of cells that were positive for CD28 (Fig. 3b), but when focusing on the samples with higher CD4^+^NKG2D^+^ T cell percentages, this number was much lower in both populations. It is interesting, however, to focus further on the differences between the populations in aggregate. We see a significant difference between patient and control samples with control CD4^+^NKG2D^+^ T cells significantly more likely to express CD28 (Mann–Whitney *U*-test; *p* = 0.008). Within the patient samples, the loss of CD28 was sharply evident in the samples that had higher levels of CD4^+^NKG2D^+^ T cells. When the patient and control samples were again divided into groups based on percentage of CD4^+^NKG2D^+^ T cells (Fig. 3c), we continued to see this division, with the loss of CD28 in both patients and controls that have high numbers of CD4^+^NKG2D^+^ T cells. Negative and statistically significant correlations between CD28 expression and percentages of CD4^+^NKG2D^+^ T cells were observed in both cancer patients (Spearman ρ = -0.859; *p* < 0.001) and healthy controls (Spearman ρ = -0.522; *p* = 0.003).

### Degranulation and cytotoxic markers tend to diminish in CD4^+^NKG2D^+^ T cells which are negative for the costimulatory CD28 molecule

**Fig. 4:**
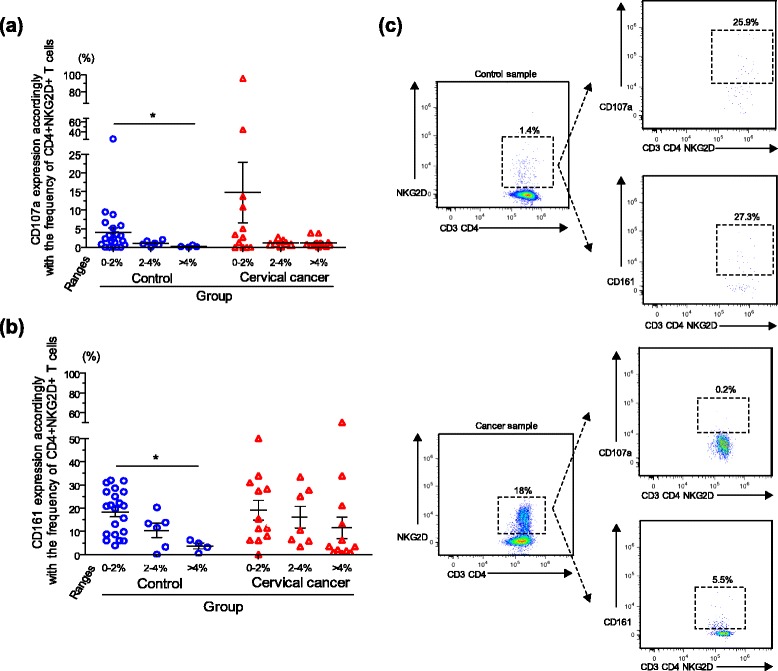
Degranulation and cytotoxic markers are diminished in CD4^+^NKG2D^+^CD28^−^ T cells. Expression of the degranulation (CD107a) and cytotoxic markers (CD161) was measured on gated CD3^+^CD4^+^NKG2D^+^ T cells in control and cervical cancer samples subdivided in turn, into the three groups based on ranges of CD4^+^NKG2D^+^ T cells (as mentioned in Results). Expressions of CD107a (**a**) and CD161 (**b**), graphed as a percentage of gated CD4^+^NKG2D^+^ T cells, were negative and statistically significant correlated accordingly with the expansion of CD4^+^NKG2D^+^ T cells (Spearman ρ = -0.480; *p* = 0.006; Spearman ρ = -0.441; *p* = 0.013, respectively) in control samples. Negative correlation in cervical cancer was not significant; however, a similar tendency to that seen in healthy donors can be also observed. Representative FACS dot plots of CD107a and CD161 expression in control (upper panel) or cancer patient (lower panel) are shown in (**c**). The expression of these markers is clearly lost in accordance with the increase of the CD4^+^NKG2D^+^ T cell population

Focusing on what appears to be a unique population of CD4^+^NKG2D^+^CD28^−^ T cells that are overrepresented in, but not exclusive, to cervical cancer patients, cells from samples with high (>4 %) levels of CD4^+^NKG2D^+^ T cells were found to be essentially negative and very low for the presence of the markers CD107a and CD161, respectively. Expression of CD107a and CD161 was graphed as a percentage of gated CD4^+^NKG2D^+^ T cells in control and cervical cancer groups subdivided, in turn, into the three groups based on ranges of CD4^+^NKG2D^+^ T cell percentages, which were characterized by the expression or lack of CD28 (Fig. 4a and b, respectively). Negative and statistically significant correlations were found in accordance with the increase of CD4^+^NKG2D^+^ T cells and the decrease of CD107a (Spearman ρ = -0.480; *p* = 0.006) and CD161 (Spearman ρ = -0.441; *p* = 0.013) expression in healthy donors. Negative correlation in cervical cancer was not significant; however, a similar tendency to that seen in healthy donors was also observed. Figure 4c depicts representative FACS dot plots of the expression of CD107a and CD161 on the gated CD3^+^CD4^+^NKG2D^+^ T cell population in a sample from a control (upper panel) or cancer patient (lower panel). The expression of these cytotoxic markers is clearly lost in accordance with the increase of the CD4^+^NKG2D^+^ T cell population. Continuing this analysis with other immunophenotypic markers, additional molecules were also evaluated within the gated CD3^+^CD4^+^NKG2D^+^ T cells. Particularly, a broad distribution of the CD45RO isoform in samples from control donors or patients, irrespective of the levels of CD4^+^NKG2D^+^ T cells, was seen (data not shown). Surface HLA-DR molecule, which is expressed *de novo* within the activation process of T cells, was heterogeneously expressed by CD4^+^NKG2D^+^ T cells from both control and patient groups (data not shown). CD158b, which is typically confined to NK cells (or some CD8^+^TCRαβ^+^ effector T cells), appeared to be mainly expressed in CD4^+^NKG2D^+^ T cells when this population was underrepresented (range 0–2 %) in both control and cervical cancer groups (data not shown).

### Plasma levels of pro-inflammatory cytokines are significantly diminished in cervical cancer patients

**Fig. 5:**
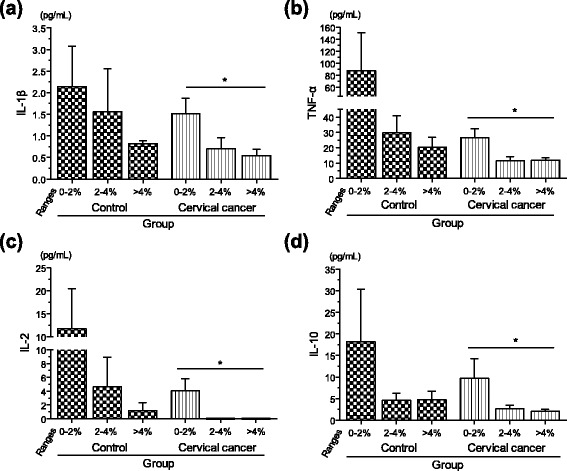
Plasma levels of pro-inflammatory cytokines are significantly diminished in cervical cancer patients. Using a commercial magnetic bead-based immunoassay, multiple cytokines were quantified in plasmas from cervical cancer patients or control women. Here again, control and cervical cancer groups were divided into the three different ranges of CD4^+^NKG2D^+^ T cells (0–2, 2–4, and >4 %). Systemic levels of IL-1β (**a**), TNF-α (**b**), and IL-2 (**c**) negatively correlated with the number of CD4^+^NKG2D^+^ T cells in cervical cancer patients (IL-1β: Spearman ρ = -0.408; *p* = 0.023; TNF-α: Spearman ρ = -0.394; *p* = 0.028; IL-2: Spearman ρ = -0.522; *p* = 0.003). The cytokine IL-10 was also negatively correlated in patient samples, accordingly with the increase of CD4^+^NKG2D^+^ T cells (Spearman ρ = -0.414; *p* = 0.021), as shown in (**d**)

Pro-inflammatory cytokines may stimulate the cell surface expression of NKG2D on CD4^+^ T cells [21]. With circulating CD4^+^NKG2D^+^ T cells frequently expanded in patients with cervical cancer, we wanted to explore the plasma cytokine profile, including pro- and anti-inflammatory cytokines, which may be involved in the expansion of this particular T cell population. Once again, control and cervical cancer groups were divided into the three different ranges of CD4^+^NKG2D^+^ T cells (0–2, 2–4, and >4 %). Interestingly, plasma levels of pro-inflammatory cytokines IL-1β (Fig. 5a), TNF-α (Fig. 5b), and IL-2 (Fig. 5c) were significantly and negatively correlated with the numbers of CD4^+^NKG2D^+^ T cells in cervical cancer samples (IL-1β: Spearman ρ = -0.408; *p* = 0.023; TNF-α: Spearman ρ = -0.394; *p* = 0.028; IL-2: Spearman ρ = -0.522; *p* = 0.003). Despite the fact that we did not find any significant correlation in control samples, a similar trend was seen as well, with the lowest values of pro-inflammatory cytokines occurring when the CD4^+^NKG2D^+^ T cell population was overrepresented (also shown in Fig. 5a, b, and c). In Fig. 5d it is notable that the cytokine IL-10, which is commonly considered as anti-inflammatory cytokine, was also negatively correlated in patient samples in accordance with the increases of CD4^+^NKG2D^+^ T cells (Spearman ρ = -0.414; *p* = 0.021). This same tendency was found in control samples, although the values were not statistically significant. Additionally, we also measured the levels of the pro-inflammatory cytokines IL-15, IL-6, IL-17, and IFN-γ, which were found to be diminished in cervical cancer patients in accordance with the expansion of the CD4^+^NKG2D^+^ T cell population; however, no statistically significant correlation was found in control or patient samples (data not shown).

## Discussion

The existence of unusual cytotoxic CD4^+^NKG2D^+^ T cells have been reported in individuals with chronic inflammatory diseases or viral infections [21, 22]; however, opposing evidence has implicated the existence of a normally-occurring CD4^+^NKG2D^+^ T cell population apparently endowed with regulatory activity in healthy individuals, and interestingly appeared to be inversely correlated with disease severity in patients with juvenile-onset systemic lupus erythematosus [23]. These confounding data lead to the speculation that two distinct CD4^+^NKG2D^+^ T cell subsets might be operating in different disease settings. Our group recently showed that in patients with cervical intraepithelial neoplasia grade-1 (CIN-1), there is an atypical expansion of circulating CD4^+^NKG2D^+^ T cells, which could be the result of a chronic exposure to viral and/or pro-inflammatory factors, and that this expansion might perhaps affect the fate of the lesion [33]. In order to extend that result to patients with more advanced stages of cervical lesions, we included in the present work a group of patients who were diagnosed histopathologically as having invasive squamous cervical carcinoma. We first stratified our results in three different ranges of CD4^+^NKG2D^+^ T cells (0–2, 2–4, and >4 %), as we previously saw that this population was not higher than 2 % in healthy controls and was increased in patients with CIN-1 (in average 3.6 % of CD4^+^NKG2D^+^ T cells) [33]; in fact, amounts still greater than 4 % were generally found in patients with persistent/recurrent disease (unpublished data). Interestingly, while only 13 % of the control samples expressed CD4^+^NKG2D^+^ T cells above the level of 4 %, a much higher percentage (39 %) of cervical cancer patients had levels of 4 % or greater of these cells. Certainly, the sole infection with HPV could explain, in part, the frequent expansion of CD4^+^NKG2D^+^ T cells seen in the cancer patients; however, we did not test for the presence of the virus (unlike our study with CIN-1 patients), since it is well known that virtually all cervical carcinoma patients (99.7 %) have shown to be HPV-DNA carriers [38].

Lack of the costimulatory molecule CD28 on CD4^+^ T cells has been reported in some chronic inflammatory diseases and elderly individuals [39–41]. Particularly, patients with advanced cervical cancer have exhibited a markedly reduced proportion of CD28^+^ cells within both CD4^+^ and CD8^+^ T cell subpopulations [42]. Perhaps one of the most striking findings in our analysis has been the fact that CD28 defines unique populations within the already rare and incompletely characterized CD4^+^NKG2D^+^ T cell population. The results shown in Fig. 3 suggest that when CD4^+^NKG2D^+^ T cells are expanded a unique CD28^−^ population predominates. If we mainly focus on cervical cancer samples, we find at least two types of patients, one with an expanded population of CD4^+^NKG2D^+^ T cells and one without. In the patients with expanded populations of CD4^+^NKG2D^+^ T cells, the CD28^−^ phenotype predominates. Given the relative numbers of these cells in the different patient groups, it is difficult to say if the patients with the expanded CD4^+^NKG2D^+^ T cells have converted the CD28^+^ cells to a negative phenotype, or if there is an expansion of other cells that simply dwarfs the absolute number of CD28^+^ cells seen in the patients with low CD4^+^NKG2D^+^ T cells. The intermediate group (2–4 % of CD4^+^NKG2D^+^ T cells) might be simply an overlapping mixture of both CD28^+^ and CD28^−^ cells. Interestingly, the same behavior was observed in samples from controls, where we can distinguish carrier and non-carriers of CD28 on the selected CD4^+^NKG2D^+^ T cell population; this finding indicates that there might exist a functional dichotomy within these subsets. Interestingly, CD4^+^CD28^−^ T cells have been frequently found in patients with autoimmune disorders and other chronic infectious diseases and it has been found that these cells are essential producers of cytolytic molecules, such as perforin and granzyme B [43–45]. In this current work, we did not identify intracellular cytolytic molecules; thus, it will be necessary to design further experiments in order to prove if these molecules are distinctive signature in any of the different CD4^+^NKG2D^+^ T cell populations, which may or may not express the costimulatory marker CD28.

In the search for immunoregulation-associated molecular markers we have interestingly found, in current ongoing experiments, in selected CD4^+^NKG2D^+^ T cells from healthy donors (with ranges between 0 and 2 % or >4 %), that there is a discrepancy regarding the expression of the master transcription factor Foxp3, with preferential expression on those T cells co-expressing both NKG2D and CD28. This suggests that a possible natural CD4^+^NKG2D^+^CD28^+^ T cell population, mostly found in healthy individuals, is endowed with immunoregulatory functions and that this regulatory role becomes altered when the population expands in pathological conditions associated with chronic inflammation, such as occurs in the case of uterine cervical cancer. More studies will be necessary, however, in order to demonstrate the lack of Foxp3 in expanded CD4^+^NKG2D^+^ T cells from cervical cancer patients.

Searching for markers to define the CD4^+^NKG2D^+^ T cell population further, we examined CD161 and CD107a. Looking deeper at this immunophenotyping, we found that the lowest levels of these cytotoxicity or degranulation markers were commonly found in samples with high CD4^+^NKG2D^+^ T cell levels. A distinction between samples from controls and patients was seen with CD161 staining where the patient samples with high CD4^+^NKG2D^+^ T cell levels expressed CD161 three fold higher than the high CD4^+^NKG2D^+^ T cell level from the controls. This may indicate the presence of activated pro-inflammatory cells (possibly Th17) in the cervical cancer patients. This idea may be plausible if we were to take into account that there is a recent report showing that CD4^+^NKG2D^+^ T cells represent a key source of IL-17 in patients with Crohn’s disease and that these cells share a Th17-related phenotype, including high expression of the cytotoxicity marker CD161 [6]. However, we may possibly have to discard this possibility due to the fact that in our analysis the lowest levels of systemic IL-17 were actually found in cervical cancer patients with high numbers of CD4^+^NKG2D^+^ T cells (data not shown); however, we will clearly need more studies to specifically detect the cytokine pattern produced by this particular population of CD4^+^NKG2D^+^ T cells in cervical cancer patients. In continuing with this and given the heterogeneity of the cancer patient group, it is not surprising that a few outlier samples expressed relatively high levels of CD107a and CD161. Interestingly, the patients that expressed starkly higher levels of CD107a (four patients) all had very low levels of CD4^+^NKG2D^+^ T cells, and three of these patients were among the five highest outliers for CD161 as well, suggesting a distinct, perhaps more cytotoxic, immune response in these patients.

On the other hand, the wide distribution of the CD45RO isoform in samples from control and patients, irrespectively of the levels of CD4^+^NKG2D^+^ T cells (data not shown), suggests a memory and/or activated state on this particular T cell population. This result is not surprising if we consider that the repetitive exposure to the same antigen seems to be a cause for NKG2D expression [4, 46]; thus, it would be reasonable to expect a memory/effector state in the CD4^+^NKG2D^+^ T cell pool. Other markers, as HLA-DR or CD158b, were heterogeneously expressed by CD4^+^NKG2D^+^ T cells from both controls and patients (data not shown), which might indicate different activation/differentiation stages within the lifespan of the CD4^+^NKG2D^+^ T cells.

Cytokine-mediated regulation of NKG2D in CD4^+^ T cells has not yet been fully understood, but, at least, there is also evidence concerning the influence of pro-inflammatory cytokines on NKG2D induction; one example is a report showing that a substantial number of peripheral and synovial CD4^+^CD28^−^ T cells in patients with rheumatoid arthritis expressed NKG2D under the influence of IL-15 and TNF-α, which endowed this T cell population with cytotoxic activity and the ability to lyse synoviocytes aberrantly expressing NKG2D ligands [21]. In our current work, however, we did not find high levels of IL-2 or TNF-α in those patients (or controls) with expanded CD4^+^NKG2D^+^ T cells; on the contrary, significant and negative correlations between the numbers of CD4^+^NKG2D^+^ T cells and levels of these pro-inflammatory cytokines were found in cervical cancer samples. At a first glance, this would seem paradoxical if we consider that a pro-inflammatory environment ought to act in favor of NKG2D expression; however, taking into consideration our preliminary results from ongoing research in healthy donors with respect to the preferential expression of Foxp3 in those CD4^+^NKG2D^+^ T cells, which in turn co-express CD28, and usually seen in the lowest range of 0–2 % (as already mentioned), it might indicate a functional divergence among the apparently different subsets of CD4^+^NKG2D^+^ T cells.

The negative correlation also found between the plasma concentrations of IL-1β and numbers of CD4^+^NKG2D^+^ T cells in cancer patients may simply be an independent variable resulting from the fact that IL-1β is typically activated in situations where TNF-α is produced. Despite the fact that we did not find significant correlations in the control group, it is important to remark that the same tendency was also observed. Data related to IL-10 are not surprising if again we consider that a hyper-secretion of this anti-inflammatory cytokine would act to regulate the production of pro-inflammatory mediators. Indeed, it has been proposed that a normally occurring CD4^+^NKG2D^+^ T cell population is tasked with the production of IL-10 and FasL and is inversely correlated with disease activity in juvenile-onset lupus [23]. Apparently, this particular T cell population, with regulatory rather than cytotoxic role, might be different than that previously described in other autoimmune disorders, such as rheumatoid arthritis [21]. However, our current study is limited by the fact that neither the cytokine profile yielded by the different subsets of CD4^+^NKG2D^+^ T cells (characterized by the expression or lack of CD28) was evaluated, nor the effect of exogenous pro-inflammatory cytokines on these cells was assessed; thus, these experiments should be planned in the future to truly reveal the meaning of the balance between CD4^+^NKG2D^+^CD28^+^ T cells and CD4^+^NKG2D^+^CD28^−^ T cells and to answer whether the CD4^+^NKG2D^+^ T cell population frequently seen in our patients promotes or discourages the growth of cervical cancer.

## Conclusions

Taken together, our results reveal the existence of two separate CD4^+^NKG2D^+^ T cell subsets defined by the co-expression or absence of CD28, the latter more likely to be present in anti-inflammatory environments and in patients with cervical cancer.

## Abbreviations

HPV: Human papillomavirus
CIN-1: Cervical intraepithelial neoplasia grade-1
NKG2D: Natural-killer group 2, member D
NKT cells: Natural killer T cells
iNKT cells: Invariant natural killer T cells
FACS: Fluorescence-activated cell sorting
FS: Forward scatter
SS: Side scatter
